# The Effect of Lesion Length on Doppler Velocities Used Routinely to Determine Carotid Stenosis Cross-Sectional Severity

**DOI:** 10.3390/diagnostics15101259

**Published:** 2025-05-15

**Authors:** Wladyslaw Dabrowski, Lukasz Tekieli, Anna Kablak-Ziembicka, Justyna Stefaniak, Karolina Dzierwa, Adam Mazurek, Piotr Paluszek, Krzysztof Zmudka, Piotr Pieniazek, Piotr Musialek

**Affiliations:** 1 Department of Cardiac and Vascular Diseases, St. John Paul II Hospital, Jagiellonian University Medical College, 31-202 Krakow, Poland; luk.tekieli@gmail.com (L.T.); kablakziembicka@op.pl (A.K.-Z.); mazurekadam@yahoo.pl (A.M.); kardio@kki.krakow.pl (P.P.); 2 Department of Interventional Cardiology, St. John Paul II Hospital, Jagiellonian University Medical College, 31-202 Krakow, Poland; k.zmudka@szpitaljp2.krakow.pl; 3 KCRI, 30-347 Krakow, Poland; 4 Data Management and Statistical Analysis (DMSA), 30-384 Krakow, Poland; jusstefa@gmail.com; 5 Department of Bioinformatics and Telemedicine, Jagiellonian University, 30-688 Krakow, Poland; 6 Cardiovascular Imaging Laboratory, St. John Paul II Hospital, 31-202 Krakow, Poland; kdzierwa@gmail.com; 7 Department of Vascular Surgery and Endovascular Interventions, St. John Paul II Hospital, 31-202 Krakow, Poland; pipal@poczta.onet.pl

**Keywords:** carotid stenosis, stenosis severity, doppler ultrasound, cross-sectional stenosis, duplex ultrasound, stenotic segment length, lesion length

## Abstract

**Background/Objective**: Transcutaneous Doppler ultrasound is a fundamental tool in evaluating carotid stenosis cross-sectional severity (CS-CSS) in clinical practice because peak-systolic and end-diastolic velocities (PSV, EDV) increase with angiographic diameter stenosis. We tested the hypothesis that lesion length (LL) may affect PSV and EDV. **Methods**: CARUS (Carotid Artery IntravasculaR Ultrasound Study) prospectively enrolled 300 consecutive patients (age 47–83 years, 64.3% men, 63.3% symptomatic) with carotid stenosis ≥50% by Doppler ultrasound considered diagnostic (corelab analyst). We correlated stenosis LL (mm) and minimal lumen area (MLA, mm^2^) with PSV and EDV. **Results**: IVUS imaging (20 MHz Volcano/Philips) was uncomplicated. As IVUS probe forward/backward movement with systole/diastole (“jumping”-related artifact superimposed on motorized pullback) restrained LL (but not MLA) determination, LL measurement was angiographic. Final data set included 293 patients/stenoses (applicable to seven angiograms unsuitable for LL measurement). Irrespective of CS-CSS, a significant LL effect on PSV and EDV occurred with LL ≥ 7 mm (n = 224/293, i.e., 76.5% study patients/lesions; r = 0.38 and r = 0.35); for MLA irrespective of CS-CCS the coefficients were r = 0.49 (PSV) and r = 0.42 (EDV); *p* < 0.001 for all. For LL and MLA considered together, the correlations were stronger: r = 0.61 (PSV) and r = 0.54 (EDV); *p* < 0.0001 for both. Combined LL and MLA effect was represented by the following formulas: PSV = 0.31 × LL/MLA + 2.02 [m/s]; EDV = 0.12 × LL/MLA + 0.63 [m/s], enabling to correct the PSV (EDV)-based assessment of CS-CSS for the LL effect. **Conclusions**: This study provides, for the first time, systematic evidence that the length of carotid stenosis significantly affects lesional Doppler velocities. We established formulas incorporating the contribution of both stenosis length and its cross-sectional severity to PSV and EDV. We advocate including stenosis length measurement in duplex ultrasound reports when performing PSV (EDV)-based assessment of carotid cross-sectional stenosis severity.

## 1. Introduction

Atherosclerotic carotid artery stenosis is an important causal factor of ischemic stroke [1,2,3,4]. Correct diagnosis of carotid artery stenosis is relevant as it triggers (1) initiation/maximization of medical therapy to reduce the stroke risk and overall atherosclerosis-associated cardiovascular risk and (2) when indicated, consideration of revascularization to optimize carotid-related stroke risk reduction on top of medical therapy [1,5,6,7]. Although there is a growing understanding of the role of plaque morphology in relation to stroke risk [5,8,9,10,11,12], according to current guidelines carotid stenosis cross-sectional severity remains the fundamental parameter in clinical decision-making [13,14,15].

Transcutaneous duplex ultrasound (DUS) is the primary diagnostic tool in establishing the presence of carotid stenosis, as it is noninvasive, contrast-free, and widely available [14,16]. As the DUS peak systolic and end-diastolic velocities (PSV, EDV) relate to angiographic diameter stenosis [17,18,19], DUS is a routine first–line technique in grading cross-sectional stenosis severity [13,14,16,20], fundamentally affecting further diagnostic and therapeutic steps in everyday clinical practice. However, no single lesion-severity classification system has yet been established. Rather, several different PSV and EDV cutoffs have been proposed in relation to specific ranges of carotid % diameter stenosis [21,22,23,24]. The disagreements in lesional velocity cutoffs [21,22,23,24] reflect the DUS poor discriminatory value in grading carotid cross-sectional stenosis severity [18,25,26,27,28]. While DUS may perform well in classifying stenoses as above or below a single degree of severity, it does not function well in subclassification of the stenosis degree [18,25,27,29]. Evaluation of velocity data for 977 carotid lesions from five studies that involved routine carotid diameter stenosis determination suggests that use of flow velocities to determine cross-sectional stenosis severity can, in a proportion of lesions, overestimate the degree of stenosis [20].

Computational fluid dynamics and experimental measurements in stenotic models indicate that hemodynamics of the stenotic carotid artery is affected by a number of parameters beyond the cross-sectional degree of stenosis [30,31,32,33,34]. Other significant variables include the stenotic segment length, cardiac output, flow rate, and flow velocity prior to the stenotic segment, blood volume and pressure, vessel compliance, and blood viscosity [30,33,34,35]. Experimental bench model data and studies in animal models consistently demonstrate that—in addition to unquestionable role of cross-sectional area of the stenotic segment—the lesion length is an important variable of both non-turbulent and turbulent flow [30,36].

**Figure 1:**
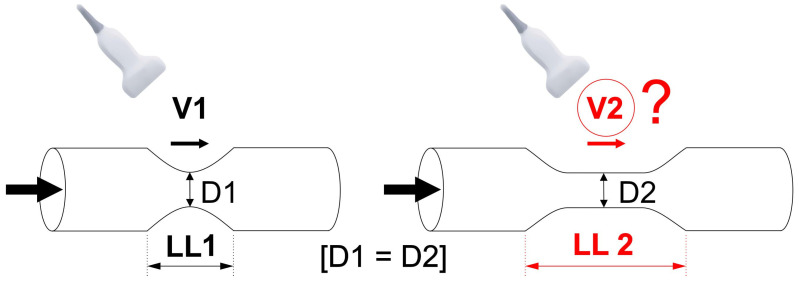
**Schematic presentation of the study hypothesis.** We hypothesized that carotid lesion length may affect the measured Doppler flow velocities in clinical practice; an effect on flow velocities beyond that of cross-sectional stenosis severity. If the hypothesis is valid, an increase in lesion length (LL2 > LL1) would correlate with a change (to maintain flow, likely—an increase) in flow velocity. This might be relevant to clinical practice as majority of DUS reports usually only include measurements of “peak systolic” (PSV) and “end-diastolic” velocity (EDV). D1—minimal lumen diameter of the stenotic segment in Lesion 1, D2—minimal lumen diameter of the stenotic segment in Lesion 2, LL—length of the lesion, V—flow velocity.

We tested the hypothesis that longitudinal length of the carotid atherosclerotic narrowing may significantly affect clinical measurements of PSV and EDV beyond the role played by cross-sectional stenosis severity (Figure 1).

## 2. Materials and Methods

### 2.1. Study Design

This was a prospective, intravascular ultrasound (IVUS)–based academic study enrolling consecutive neurologically asymptomatic or symptomatic patients referred with carotid artery de novo stenosis of at least “50%” in the context of potential revascularization (Carotid Artery IntravasculaR Ultrasound Study, CARUS). The target population of 300 participants was recruited over a period of 19 months. Patients with contraindications to angiographic imaging or with highly calcified lesions that precluded reliable assessment of velocities [37,38,39] as per agreement of two experienced ultrasonographers were excluded. Those with chronic kidney disease with glomerular filtration rate < 30 mL/min were also excluded because catheter angiography imaging could require several projections for optimal lesion visualization. Furthermore, the presence of ≥50% contralateral stenosis was an exclusion criterion because it may affect the index vessel flow velocities particular for contralateral stenoses > 70% [40,41]. Contralateral stenoses 50–70% may exert only minimal effects on ipsilateral flow velocities while the effect of those <50% is negligible [41,42]. Patients were considered symptomatic when an independent neurologic consultation indicated a history of ipsilateral hemispheric (transient ischemic attack or stroke) or retinal (amaurosis fugax, retinal stroke) symptoms within the preceding six months in the presence of atherosclerotic carotid stenosis. In symptomatic patients, in case of bilateral internal carotid artery stenosis, the symptomatic vessel was the study artery. DUS and subsequent quantitative catheter angiography (QA) and IVUS were performed during index hospitalization, within 3 days from one another.

### 2.2. DUS Examination

DUS scanning was performed in a certified vascular ultrasound imaging lab, using a Toshiba Aplio PowerVision ultrasound machine (Toshiba Medical Systems Co., Ltd., Tokyo, Japan) equipped with a 4–11 MHz linear-array transducer, by one of two experienced operators working together on a daily basis. In case of doubt, the other DUS operator was consulted, and an agreement was reached. The Doppler waveform was obtained with an angle of insonation equal to 60°; angles between 45° and 60° were considered in case of anatomic constraints. The carotid artery was sampled through the region of stenosis completely until the distal end of the plaque was visualized, to ensure that the site of greatest velocity had been captured. The highest flow velocities in systole and end-diastole (PSV and EDV) were recorded for further analysis. The quality of DUS recording had to be of passed as diagnostic by a senior corelab analyst. DUS examination preceded catheter angiographic and IVUS examination by 1–3 days (median 1 day).

### 2.3. Quantitative Catheter Angiography (QA)

**Figure 2:**
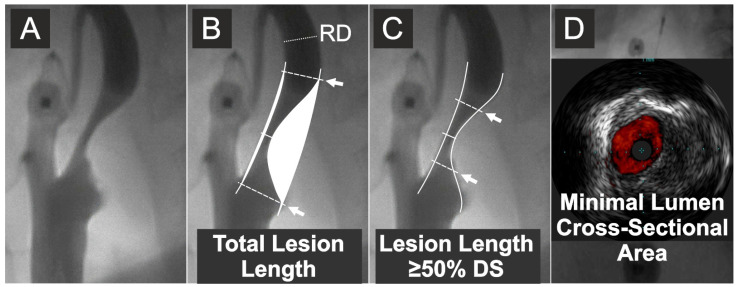
**Quantitative measurements of the lesion length and IVUS evaluation of minimal lumen area.** (**A**) Carotid artery catheter angiography in a symptomatic lesion. (**B**) Quantitative angiography measurement of the total lesion length and stenosis severity by % diameter stenosis. (**C**) Quantitative angiography measurement of the length of stenotic segment with cross-sectional severity of at least 50% diameter stenosis (DS). (**D**) IVUS evaluation (in this case, under proximal neuroprotection by transient flow reversal) of the minimal lumen cross-sectional area. Arrows indicate, respectively, total lesion length (**B**) and the length of stenotic segment with cross-sectional severity of ≥50% diameter stenosis (**C**). RD is the reference diameter. Diameter stenosis (%) was calculated according to the NASCET method [43]; cf., Supplementary Figure S1.

After obtaining transfemoral or transradial access, unfractionated heparin was routinely administered at the dose of 5000 IU. In case of cerebral protection device use or further proceeding to intervention, the heparin dose was further titrated to achieve an activated clotting time of at least 250 s. Selective digital angiography of the index carotid artery was performed using Coroscop or Axiom Artis Zee angiograph (Siemens AG, Munich, Germany) in multiple (four to seven, median four) angulated projections to identify the narrowest lumen diameter while minimizing foreshortening and avoiding an overlap of side branches. The view where the stenosis was tightest was used for quantitative cross-sectional measurements (Quantcor QA v5.0, Siemens) performed consistent with a core laboratory standard operating procedure. QA measurements, including reference diameter (RD—determined as per NASCET [43]), minimal lumen diameter (D_min_), and diameter stenosis (DS) were performed offline by agreement of two Angiographic Core Lab analysts and were then verified by an angiographic corelab supervisor. Percent diameter stenosis was computed according to the North American Symptomatic Carotid Endarterectomy Trial (NASCET) method as [(RD-D_min_)/RD] × 100%, where RD is the reference diameter and D_min_ is the minimal lumen diameter [43]. However, the blood flow velocity in the stenosed artery is known to be dependent on the area cross section rather than on the lumen symmetry [44]. Because of the limitations of circumferential MLA (QA-MLA) estimation from a planar D_min_ measurement (π × (D_min_/2)^2^ formula), relevant particularly to non-circular lumens (example in Figure 2), a contrast column density–based (i.e., denistometric) evaluation of MLA (QA_DENSITOM_-MLA) [45] was performed in addition to the MLA estimation from planar images (MLA = π (D_min_/2)^2^). While intravascular ultrasound (IVUS) was used for its precise determination of MLA, our pilot work demonstrated that the systolic/diastolic longitudinal movement of the IVUS probe (a “jumping”-related artifact, superimposed on automatic pullback) restrains the LL (but not MLA) measurement while QA performs well in longitudinal measurements [46]. Thus, angiographic LL determination (performed in the projection showing the greatest lesion length) was used (Figure 2). With precise lesion-length measurement considered unfeasible in seven angiograms, the final data set included 293 patients/lesions. Angiographic and ultrasound measurements were taken in maximal diastole of the vessel.

### 2.4. Intravascular Ultrasound

Because flow velocity is inversely related to cross-sectional area of the stenotic segment [29], accurate determination of the cross-sectional area is fundamental to dissect out the lesion-length contribution to flow velocity. As planar QA is routinely focused on longitudinal rather than cross-sectional imaging, intravascular ultrasound (IVUS) was used for precise MLA measurements [45,46,47,48]. Consistent with our prior experience [49,50], the decision to use a neuroprotection device for IVUS imaging was based on the lesion morphology, stenosis severity, and the presence/absence of a history of ipsilateral clinical symptoms or an ipsilateral asymptomatic cerebral infarct, and it was left to the operator performing the case.

A commercially available rapid-exchange IVUS transducer (3.5 F, scanner diameter 1.15 mm, Eagle Eye Gold or Platinum, Philips Volcano, San Diego, CA, USA) was introduced to the index carotid artery over a 0.014-inch coronary guidewire (in case of unprotected imaging or imaging under proximal cerebral protection) or, in case of distal embolic protection device use, over the wire of the protective filter. At least two IVUS runs with automatic motorized pullback were performed with the speed of 0.5 mm/s. ChromaFlo application (Philips Volcano) was routinely used in one IVUS run to improve delineation of the interface between the lumen and the vessel wall or atherosclerotic plaque (Figure 2D) [50]. In addition, a very slow manual pullback was performed in order not to miss the minimal lumen spot [50]. IVUS measurements of MLA were performed at maximal vessel diastole using QIvus software (v.2.0, Medis Medical Imaging Systems, Leiden, The Netherlands). Precise tracking of the lumen border—using manual correction whenever necessary—was performed on a frame-by-frame basis. QIvus software lumen area calculation was performed, taking into consideration the delineated lumen shape, including elliptic-like (example in Figure 2D) or any other non-circular lumen shapes. Lumen area measurement in the MLA frame was used for further analysis. Reference lumen diameter was taken as the maximal lumen diameter in the reference segment in diastole. IVUS measurements were performed by agreement of two IVUS core laboratory analysts with >10 years of experience in carotid IVUS evaluation. Consistent with a standard operating procedure, IVUS measurements were further approved by the IVUS core laboratory supervisor. Analysts performing DUS, QA, and IVUS measurements were blinded to one another.

The study was conducted according to the guidelines of the Declaration of Helsinki and approved by the Jagiellonian University Medical College Ethics Committee. Informed consent was obtained from all participants involved in the study.

### 2.5. Stenosis Parameters

**Figure 3:**
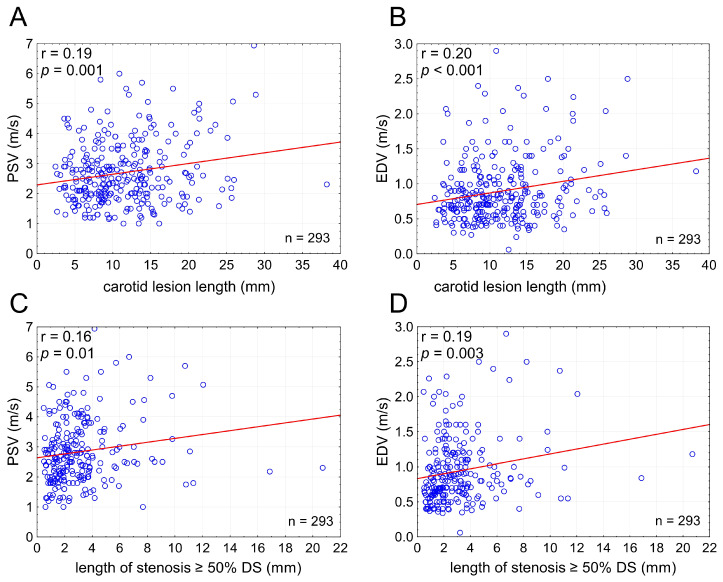
**Carotid lesion length affects flow velocities irrespective of cross-sectional stenosis severity.** Graphs present relationship between the stenotic segment length (total lesion length in (**A**,**B**) and the length of stenotic segment with ≥50% DS ((**C**,**D**); cf., Figure 2) and PSV (**A**,**C**) or EDV (**B**,**D**). PSV—peak systolic velocity, EDV—end-diastolic velocity, DS—(angiographic) diameter stenosis, n—number.

**Figure 4:**
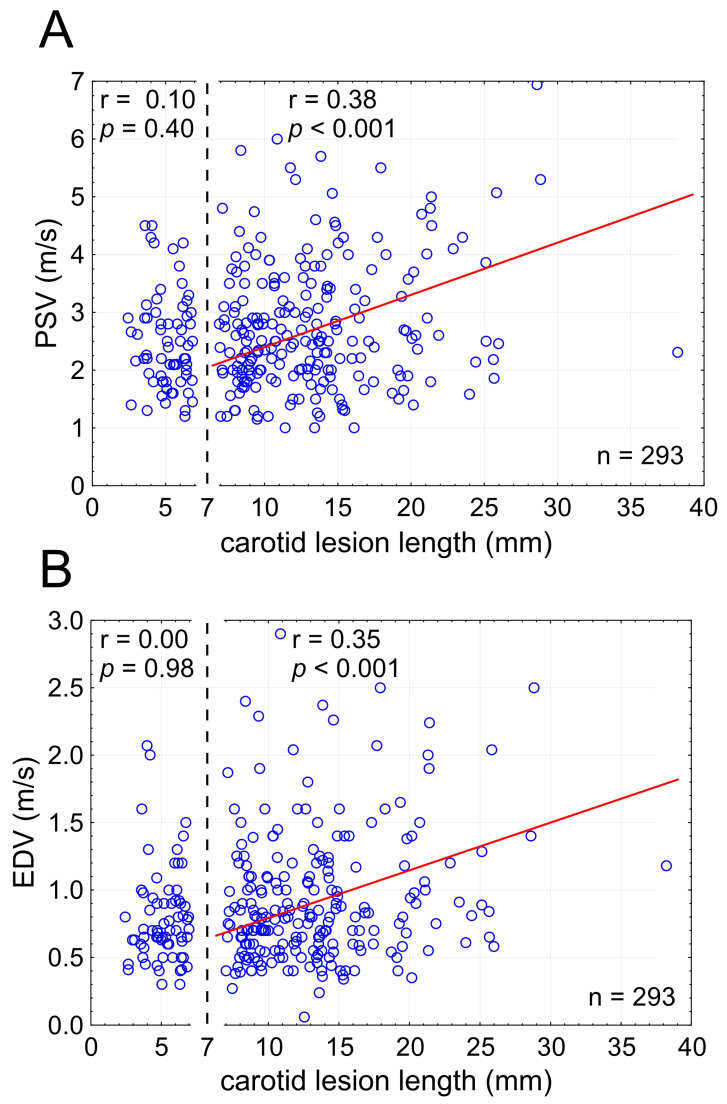
**Stenotic segment length cutoff for the effect on lesional flow velocities ((A)—PSV; (B)—EDV).** Note that the effect on flow velocities is statistically significant for carotid lesion lengths (LL) of at least 7 mm. In the present consecutive patient sample, only a minority of lesions (n = 69; 23.5%) showed LL < 7 mm, consistent with the LL effect on flow velocities applicable to a majority (>75%) of carotid stenotic lesions. LL—total lesion length, PSV—peak systolic velocity, EDV—end-diastolic velocity, n—number.

**Figure 5:**
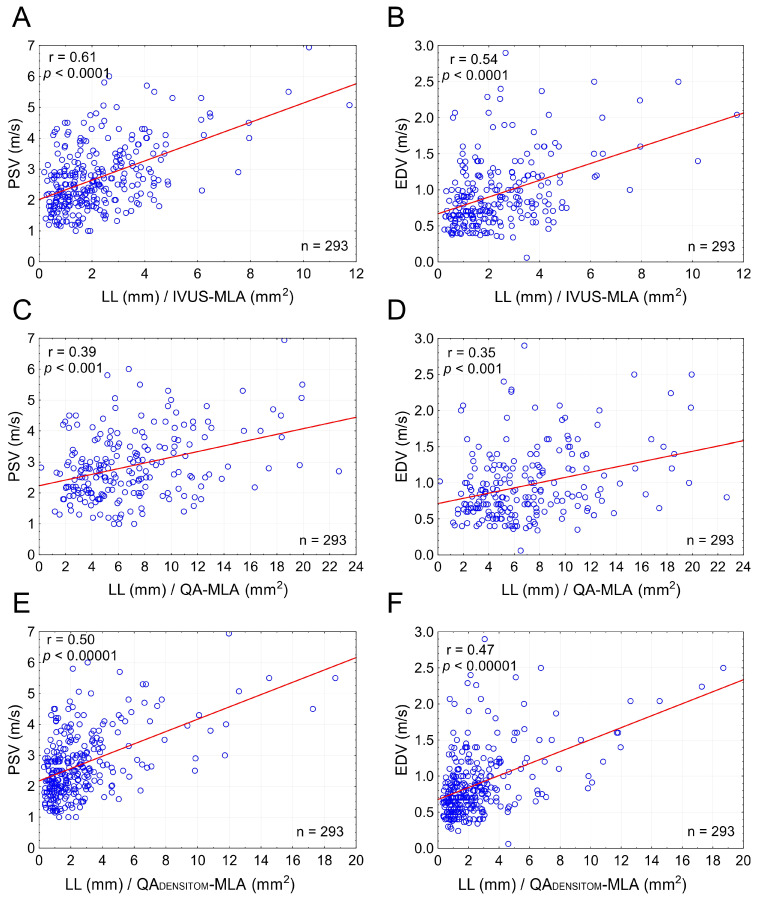
**Combined effect of lesion length and cross-sectional stenosis severity on flow velocities in carotid stenotic lesions.** Graphs on the left (**A**,**C**,**E**) show a combined effect of LL and MLA on PSV whereas those on the right (**B**,**D**,**F**) demonstrate a combined effect of LL and MLA on EDV. In (**A**,**B**) the LL is indexed to MLA by IVUS; in (**C**,**D**) the MLA is estimated from conventional quantitative angiography measurements, whereas in (**E**,**F**) the MLA is automatically determined from the minimal contrast column density in the stenotic segment in relation to that in the reference segment. Note that the correlation coefficients in (**E**,**F**) nearly reach those in (**A**,**B**), consistent with a greater accuracy of MLA determination via the automatic densitometric measurement rather than estimation from a conventional (planar) contrast angiography. See text for details. PSV—peak systolic velocity, EDV—end-diastolic velocity, LL—total lesion length, IVUS-MLA—intravascular ultrasound minimal lumen area. QA-MLA—minimal lumen area estimation from conventional (planar) quantitative angiography; QA_DENSITOM_-MLA—quantitative angiographic densitometric (contrast column density-based) minimal lumen area, n—number.

The following parameters were analysed in the context of investigating relationship(s) between the stenotic flow velocities and characteristics of the narrowing: (1) PSV and EDV (m/s), (2) the carotid stenotic segment total lesion length (LL, mm) (Figure 2), (3) length of the carotid stenotic segment with stenosis severity of at least 50% DS (mm); (Figure 2); (4) IVUS-MLA (mm^2^) (Figure 2); (5) QA-MLA (mm^2^); (6) QA_DENSITOM_-MLA (mm^2^). The length of stenotic segment with stenosis severity ≥50% DS was also analysed in order to indirectly assess whether the hemodynamic phenomena at the lesion entrance/exit play any major role in LL effect on stenotic peak flow velocities.

### 2.6. Data Presentation and Statistical Analysis

Categorical variables are presented as numbers and percentages. Continuous variables are expressed as median and quartiles (Q1–Q3). Correlation between lesional flow velocities (PSV, EDV) and the stenosis characteristics (total LL, LL ≥ 50% DS, IVUS-MLA, QA-MLA, and QA_DENSITOM_-MLA) is presented as a correlation coefficient (‘r’; ranging from 0 [no correlation] to 1.0 [perfect correlation]). Mann–Whitney U test was used to assess differences in the distributions between the two groups. Regression analysis was performed for the stenosis combined length and cross-sectional severity effect against PSV and EDV. Probability values < 0.05 were considered statistically significant.

## 3. Results

### 3.1. Characteristics of Patients and Index Lesions

The majority of the 293 patients (63.8%) were clinically symptomatic. Table 1 shows clinical characteristics of the study subjects whereas characteristics of the study lesions is provided in Table 2. Median PSV was 2.5 (1.9–3.3) m/s; EDV 0.9 (0.6–1.2) m/s (range from 0.97 m/s to 6.94 m/s for PSV and from 0.23 m/s to 2.91 m/s for EDV). IVUS determination of MLA was protected in 184 (62.8%) stenoses (filters—48.1%, transient flow reversal l devices—14.7%) whereas 109 (37.2%) lesions were imaged with IVUS in absence of a cerebral protection device. Catheter angiography and IVUS imaging were uncomplicated.

The carotid stenotic segment total LL was from 2.44 to 38.21 mm; median 10.46 (7.23–14.35) mm. Median minimal lumen diameter by QA was 2.02 (1.72–2.35) mm and median reference diameter was 4.95 (4.69–5.19) mm, resulting in a median DS of 59% (54–67%). Densitometric MLA evaluation showed values systematically greater than the circumferential estimation of MLA from angiographic D_min_ (median 5.9 vs. 3.3 mm^2^; *p* < 0.001). The densitometric MLA values were close to those determined by IVUS (median IVUS-MLA 6.1 mm^2^; *p* = 0.37). D_min_ by IVUS ranged from 1.2 mm to 4.2 mm; IVUS-MLA was 1.3 mm^2^–17.4 mm^2^.

### 3.2. Correlation of the Stenosis Individual Characteristics with Flow Velocities

LL showed a positive, moderate but statistically highly significant, correlation with PSV (r = 0.19) and EDV (r = 0.20); *p* = 0.001 and *p* < 0.001, respectively. With an increase in LL there was an increase in PSV and EDV irrespective of cross-sectional stenosis severity (Figure 3). The relationship between PSV/EDV and lesion length was slightly weaker for lesion length taken as the length of stenotic segment with ≥50% DS than that for the total stenotic segment length (Figure 3), indicating that total LL is a more powerful driver of the lesion length effect on PSV/EDV than length of the stenotic segment exceeding 50% of the lumen diameter.

IVUS-MLA, when considered irrespective of LL, correlated with PSV and EDV with coefficients greater than those for LL alone (r = 0.49 and r = 0.42, *p* < 0.001 for both), indicating that the individual effect of cross-sectional stenosis severity on PSV/EDV is greater than that of LL in isolation. ROC analysis, taking the cross-sectional area stenosis ≥75% by IVUS as reference, demonstrated diagnostic accuracy of uncorrected PSV slightly greater than that of uncorrected EDV, with the PSV cutoff of 2.58 m/s (area under the curve 0.77 (95% CI 0.72–0.83), sensitivity 0.74, specificity 0.71, positive predictive value 0.64, negative predictive value 0.80). The corresponding values for EDV were the following: cutoff 0.75 m/s, area under the curve 0.74 (95% CI 0.68–0.80, sensitivity 0.77, specificity 0.61, positive predictive value 0.57, negative predictive value 0.80).

### 3.3. Threshold for the Lesion-Length Effect on PSV and EDV

Candidate LL thresholds from 3 to 20 mm were tested in evaluating whether there is a cutoff for the LL effect on PSV and EDV. This revealed a significant LL effect on PSV/EDV for lesion lengths ≥ 7 mm (Figure 4) indicating relevance of the LL effect on flow velocities to majority of carotid stenoses (224/293 lesions, i.e., 76.5% in this series).

### 3.4. Combined Effect of Lesion Length and Cross-Sectional Stenosis Severity on PSV and EDV

**Figure 6:**
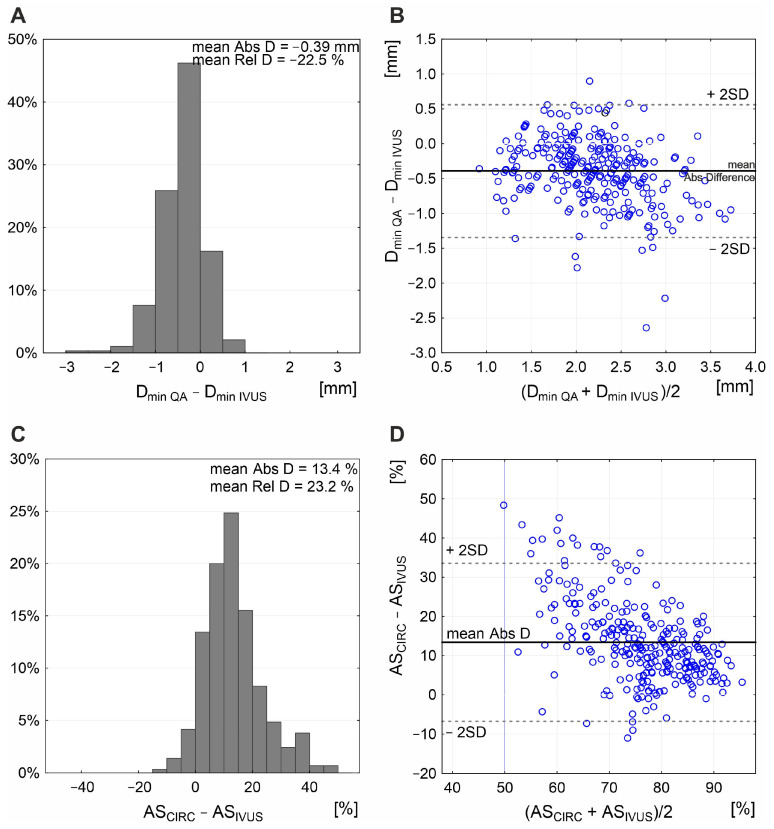
**Relationship between planar angiographic and IVUS measurement of D_min_ and cross-sectional stenosis severity.** Histogram (**A**) and Bland–Altman presentation (**B**) demonstrate systematic underestimation of D_min_ by angiographic measurement, with mean absolute difference (Abs D) of −0.39 mm and mean relative difference (Rel D) of −22.5%. This, along with inability of the planar angiogram–based estimation to correct for any non-circular lumen (example in Figure 2D), results in a systematic overestimation of cross-sectional stenosis severity (mean Abs D in area stenosis of 13.4%; (**C**) with a rather wide data scatter on Bland–Altman analysis (**D**). AS_CIRC_—circular estimation of cross-sectional carotid stenosis severity based on angiographic measurement of reference diameter and minimal lumen diameter, AS_IVUS_—intravascular ultrasound (IVUS) measurement of area stenosis (AS), QA—quantitative angiography, D_min_—minimal lumen diameter.

The combined effect of LL and cross-sectional stenosis severity was best represented by the LL/cross-sectional stenosis severity ratio. Figure 5 shows the integrated effect of LL and cross-sectional stenosis severity on flow velocities. The combined effect of LL and cross-sectional stenosis severity on peak flow velocities (PSV and EDV) was stronger than the individual effect of LL or cross-sectional stenosis severity (compare Figure 5 vs. Figure 3). The highest correlation was seen for LL combined with cross-sectional stenosis severity expressed as IVUS-MLA (r = 0.61 for PSV and 0.54 for EDV; *p* < 0.0001 for both; Figure 5A,B). Correlation coefficients were lower when replacing the IVUS-MLA with QA circumferential estimation of MLA (Figure 5C,D), consistent with a lesser accuracy of lumen estimations based on planar angiographic measurements (Figure 6). Although angiographic D_min_ correlated with D_min_ by IVUS (r = 0.71, *p* < 0.001), there was a systematic underestimation of D_min_ by QA (Figure 6A,B), translating into a systematic error of cross-sectional stenosis severity determination by QA in relation to IVUS (despite a reasonable correlation of the 2 measurements; r = 0.60, *p* < 0.01; histogram and Bland-Altman plot in Figure 6C,D, respectively). In contrast to planar measurements of the residual lumen, QA_DENSITOM-MLA_ performed only slightly less accurately than IVUS-MLA in the relationship between flow velocities and combined effect of lesion length or cross-sectional stenosis severity (Figure 5E,F versus Figure 5A,B). This is consistent with improved accuracy of angiographic MLA estimation when using automatic analysis of contrast column density at the tightest stenosis section (r = 0.82 and *p* < 0.001 for IVUS-MLA vs. QA_DENSITOM_-MLA, mean MLA by IVUS 6.1 mm^2^, mean MLA by QA_DENSITOM_ 5.9 mm^2^, mean relative difference only 7.4%).

### 3.5. Flow Velocity Formulas Integrating the Effect of Lesion Length and Cross-Sectional Stenosis Severity

By regression analysis, the combined effect of LL and cross-sectional severity on lesional Doppler velocities was represented by the following best-fit equations:

**PSV = 0.31 × LL/MLA + 2.02 [m/s]** (1)

and

**EDV = 0.12 × LL/MLA + 0.63 [m/s]** (2)

where LL is lesion length in mm and MLA is minimal lumen area in mm^2^, with the formulas operational for PSV > 2.02 m/s and EDV > 0.63 m/s.

Because **%DS = [(RD − Dmin)/RD] × 100%,** once the lesional flow velocities and LL are established, the %DS can be estimated using the above equations, as

$$Dmin=\sqrt{\frac{1.24*LL}{\pi *\left(PSV-2.02\right)}}\;[mm]\;\;\;\;\;when\;assessed\;from\;PSV$$

and

$$Dmin=\sqrt{\frac{0.48*LL}{\pi *\left(EDV-0.63\right)}}\;[mm]\;\;\;\;\;when\;assessed\;from\;EDV$$

## 4. Discussion

The fundamental, new finding from this study is that the *length* of carotid stenotic segment affects lesional Doppler velocities that are routinely used to assess the *cross-sectional severity* of carotid stenosis; a key parameter in clinical decision-making. The greater the lesion length, the greater PSV and EDV; an effect that becomes significant with lesion length ≥7 mm. We thus advocate including stenosis length measurement in DUS reports and taking the lesion length effect into account when performing PSV/EDV-based assessment of carotid stenosis cross-sectional severity.

### 4.1. Carotid Stenosis Cross-Sectional Severity: A Classic Measure of Stroke Risk

Randomized trials of asymptomatic carotid stenosis, Asymptomatic Carotid Atherosclerosis Study (ACAS [51] and Asymptomatic Carotid Stenosis Trial-1 (ACST-1 [52]), demonstrated absolute stroke reduction absolute 5.9% and 5.2%, respectively, over 5 years, with major/disabling stroke reduction by 2.6% in both trials. Further evidence from ACST-1 showed that the effect of carotid revascularization is maintained for at least 10–15 years, and it is maintained in patients on “triple” medical therapy, including statin, anti-platelet agent, and anti-hypertensive agent [1]. It is important to understand that contemporary pharmacologic therapy—even when maximized—may reduce, but it does not abolish, stroke risk in relation to carotid artery stenosis [1], and patients on optimized (by today’s criteria) medical therapy continue to present with carotid-related strokes as demonstrated in randomized trials and registries of acute stroke [1,2,3,53].

### 4.2. Interpretation of DUS Velocities: What This Study Adds

For nearly 40 years, Doppler ultrasonography stenotic flow velocities—PSV and EDV—have been in routine clinical use to assess cross-sectional severity of carotid atherosclerotic narrowings in the context of clinical decision-making [21]. Multiple studies, however, have pointed to poor discriminatory value of PSV and EDV in the grading of cross-sectional stenosis severity [18,25,26,27,28,29]. This constitutes an important limitation of the Doppler technique when used to determine cross-sectional severity of carotid stenosis. We found that that the length of carotid stenosis, when ≥7 mm, exerts a significant effect on stenotic flow velocities irrespective of cross-sectional stenosis severity—a critical parameter in clinical decision-making [13,14,15,16]. The greater the length of the narrowing, the greater PSV and EDV (Figure 4). This real-life finding, applicable to >75% patients with carotid stenosis evaluated for further management following the initial Doppler examination in this study, is consistent with the effect of LL on stenotic flow velocities demonstrated in experimental flow model studies and by computational fluid dynamics [29,30,31,32,33]. The results from the present study show that although the LL effect on flow velocities is weaker than that of cross-sectional lesion severity, it is statistically significant (Figure 4) and functionally relevant (Graphical Abstract, for a detailed description of Graphical Abstract see the Supplementary Materials). We further show that the combined effect of LL and cross-sectional stenosis severity on PSV and EDV is stronger than the roles played individually by LL and cross-sectional stenosis severity (Figure 5). Our finding of the LL effect on EDV is consistent with the postulated clinical role of implementing EDV (beyond PSV) in routine DUS analysis [54]. Finally, we established simple formulas that include the combined contribution of LL and cross-sectional stenosis severity to the DUS-captured values of lesional PSV and EDV, enabling to correct the PSV (EDV)-based assessment of cross-sectional stenosis severity for the effect of LL on flow velocities.

### 4.3. Cerebrovascular Circulation: Role of the Circle of Willis in Providing Collateral Flow

Cerebrovascular circulation relies on four major feeding vessels: two carotid and two vertebral arteries [55,56]. On the base of the brain, connecting all major feeding vessels is the circle of Willis, which is constitutes the primary collateral system [55,56]. Microvascular intracranial collaterals consisting of leptomeningeal and subcortical collaterals make the secondary collateral system with the addition of extracranial–intracranial vascular anastomoses as the third system [55,56]. The circle of Willis is a ring of interconnected medium-sized arteries located at the base of the brain. It connects the anterior and posterior circulations and the left and right hemispheres. The circle of Willis is considered the most important collateral network that connects the major feeding arteries of cerebral circulation. The circle of Willis can provide immediate diversion of blood flow in the case of an acute occlusion of a parent vessel. An important caveat is that the circle of Willis has many possible variations that significantly affect the capacity of the circle of Willis to provide collateral pathways of blood flow [55,56]. A complete circle of Willis is present in only 25% of cases. Incomplete anterior half is found in 57% of cases whereas incomplete posterior half in 3% of cases. Incomplete anterior and posterior portions are present in 15% of cases [57]. In general, the patients with a complete circle of Willis exhibit lower stroke severity compared to those with incomplete circle of Willis [57]. Although internal carotid artery occlusion can (in some patients) occur without cerebral damage, in many cases there is a risk of developing low-flow infarcts when the collateral blood supply is not sufficient to maintain adequate cerebral perfusion [58]. In the least extreme cases, the internal carotid artery supplies only the middle cerebral artery; in the most extreme cases, the internal carotid artery supplies the ipsilateral posterior cerebral artery, middle cerebral artery (and arteries arising from it), as well as both ipsilateral and contralateral anterior cerebral artery. With ~90% carotid-related ischaemic strokes being embolic and ~10% purely hemodynamic [59], functionality of the circle of Willis plays an important role in stroke outcome, whether mechanistically hemodynamic or embolic [60,61]. In carotid artery disease, the contralateral carotid artery and basilar artery may act as an important intracranial collateral to supply hypoperfused middle cerebral artery territories [62,63]. Recent transcranial Doppler ultrasonography analysis revealed a low prevalence of functional collateral cerebral circulation in the circle of Willis in patients with severe carotid artery stenosis and recent stroke [64], consistent with the presence of a compromised circle of Willis by CT-angiography in >80% patients undergoing carotid revascularization [65]. Although robust collateral networks are considered an independent predictor of better functional outcomes [57], collateral failure is an important factor of cerebral infarct progression [66]. Indeed, any functional recruitment of anatomic collateral is variable, and it may be time-dependent in relation to exhaustion of autoregulatory vasodilatation and arterial blood pressure, leading to infarct progression [66,67,68]. This may be particularly relevant to carotid-related strokes, where anatomic collaterals may not be sufficiently protective as the stroke occurrence may represent a functional exhaustion of the collateral system [56].

### 4.4. Duplex Sonography: Its Advantages and Central Role in Determining Patient Management Pathway

Extracranial duplex sonography is a principal guideline-supported tool in stroke medicine [69]. DUS simultaneously visualizes the anatomy of vessels, the causes of luminal stenosis or occlusion, and the neighboring structures. Unlike the nowadays increasingly used static imaging methods (computed tomography/computed tomography angiography, CT/CTA, and magnetic resonance imaging/magnetic resonance angiography), the ultrasonic methods allow for the real-time assessment of hemodynamics, and also the straightforward diagnosis of distinct extracranial vessel pathologies such as stenosis, mobile thrombi, dissection, carotid web, or arteritis. DUS can also be used in pre-hospital emergency diagnostics in acute stroke and in elective assessment of carotid plaque vulnerability/neovascularization by contrast-enhanced ultrasound or advanced microvascular imaging [69]. Effective prevention is inarguably the best option for reducing the individual and societal burden of stroke, and appropriate screening tools are integral to early detection and prevention of carotid-related stroke [1,6,70]. DUS is safe, affordable, widely available, and contrast- and radiation-free [59]. Other fundamental advantages of DUS are its noninvasiveness, easy repeatability, and mobility [51]. These enable point-of-care use, facilitating patient allocation and treatment decisions [51].

### 4.5. Inaccuracy of DUS Classic Interpretation

Despite its widespread use and proven diagnostic value, DUS has several important limitations. These include the DUS poor performance in subclassification of carotid stenosis cross-sectional severity; the key parameter in guideline-based clinical decision-making [13,14,15,16,44]. Evidence shows that the accuracy of non-invasive imaging of carotid stenosis severity is greatly overestimated in clinical practice [71]. Misclassification of stenosis severity with DUS will lead to some patients being operated on unnecessarily and others being denied appropriate revascularization to prevent carotid-related stroke [72]. Results from the present study indicate that at least part of the ‘inaccuracy’ of DUS velocities in determining cross-sectional stenosis severity may arise from the effect of stenotic segment length on PSV and EDV (Figure 3, Figure 4 and Figure 5).

### 4.6. Stenotic Flow Velocity Determinants: From Fluid Dynamics Theory, Through Experimental Models, to Humans

Flow velocity is driven by the pressure gradient [73]. Fluid mechanics indicate that the flow through a stenosis is non-turbulent through the body of the stenosis but becomes turbulent at the exit of the stenosis [74]. Thus, the total pressure gradient, driving the flow velocity, is determined by the sum of gradients caused by the non-turbulent intrastenotic flow and the turbulent exit flow. The variables that determine the pressure gradient relate differently to the non-turbulent and turbulent flow [75]. The pressure gradient due to non-turbulent flow is lineary proportional to the flow rate, whereas the gradient due to turbulent flow is proportional to the square of flow rate [75]. In 1978 Lipscomb and Hooten [30] published their seminal paper on the effects of lesion dimensions on flow in an experimental model of stenosis that involved modulation of the stenosic segment length and diameter. The kinetic energy possessed by a fluid is proportional to the product of its density and the square of its velocity. At normal values of lumen diameter and flow rate, the velocity is low enough to permit the kinetic energy to be disregarded. However, when the velocity increases to pass through the decreased cross-sectional area of a stenosis, pressure is converted to kinetic energy, and in laminar flow the velocity in the center of the flow stream is twice the average velocity. As the fluid passes through the exit of the stenosis, the velocity of the fluid decreases. At this point, the kinetic energy possessed by the high-velocity flow may be totally or partially dissipated in turbulence [75,76]. The pressure gradient due to non-turbulent flow is directly proportional to blood flow and length of stenosis. The component of the pressure gradient due to turbulent flow occurs when the high-velocity flow within the stenosis meets the slow-velocity flow downstream [76]. Lipscomb and Hooten [30] found that although a relative change in diameter has a greater effect on the pressure gradient than a change of the stenosis length, the length of the stenotic segment is an important variable of both non-turbulent and turbulent flow [30].

### 4.7. Hemodynamic Relevance of the Lesion Length: Coronary Artery Cutoffs

Feldman and colleagues [36] confirmed the importance of LL in vivo by manipulating the stenotic segment length (1 to 15 mm) and its cross-sectional severity (60% and 90%) in the right coronary artery in dogs. While short 60% narrowings had no significant hemodynamic influence, increasing the stenotic segment length to 10 or 15 mm consistently affected pressure gradients and flow, so that the effect of 15 mm-long 60% stenosis was similar to that of a short 90% stenosis [36]. These experimental findings in the animal model were subsequently confirmed by invasive measurements in humans, indicating a coronary stenotic segment cutoff of ≥10 mm in length for a hemodynamic significance of stenoses 50–70% in diameter by QA [77]. Subsequently, a study in 79 coronary lesions of 30% to 60% DS found a correlation coefficient of −0.42 (*p* < 0.001) for the relationship between functional flow reserve vs. LL, indicating a progressive reduction in functional flow reserve with an increase in LL [78]. In the absence of a difference in mean diameter stenosis (*p* = 0.78), the mean LL in hemodynamically significant lesions was 12.4 mm vs. 7.8 mm LL in lesion lengths with functional flow reserve ≥ 0.8 (*p* = 0.002) [78]. Kristensen et al. [79] indicated coronary LL cutoff of ≥10 mm as a strong determinant of an abnormal coronary functional flow reserve. The ≥10 mm coronary LL cutoff, above which LL plays a significant hemodynamic role, is close to the ≥7 mm length threshold that we have presently determined for functional significance of carotid lesions (Figure 4). More recently, several studies correlating coronary lesion characteristics by CTA with invasive functional flow reserve [80,81,82,83,84] demonstrated that LL taken together with cross-sectional severity (expressed as MLA, minimal lumen diameter, or area stenosis) are strong predictors of abnormal coronary functional flow reserve.

### 4.8. The Coronaries vs. Carotids: Fundamental ‘Downstream’ Differences

For a variety of reasons (including, but not limited to, differences in flow patterns and vessel size, the differences in potential collateral alternative flow distal to the stenosis in the coronary myocardial circulation vs. the cerebrovascular circulation), evidence from the coronary tree may not be directly transferrable to the carotid arteries. Furthermore, the vessel size differences are important in the computational analysis, which depicts blood as a Newtonian fluid with a viscosity representing the relationship between shear rate and shear stress as a linear function whereas blood, with a high concentration of particles, is not a Newtonian fluid; this difference becomes particularly important in small conduits.

Hemodynamic effects of carotid stenosis are assessed by cerebrovascular flow reserve with transcranial Doppler measurement of ipisilateral middle cerebral artery flow velocity using voluntary breath holding or breathing elevated concentrations of CO_2_ [85,86,87,88]. Exhausted CO_2_ reactivity correlates with the presence of carotid-related hemodynamic infarctions [89,90]. Hemodynamic effect of anatomic stenosis severity may constitute an important determinant of carotid-related stroke, whether hemodynamic or embolic [87]. Stent-assisted correction of severe carotid stenosis results in ipisilateral middle cerebral artery flow velocity increase by >25% [91].

### 4.9. Carotid Lesion Length: Why a Missed Determinant of Stenotic Flow Velocities?

As we have not evaluated the reactivity of cerebral vessels but, rather, performed the analysis of LL as a contributor to PSV and EDV, the results in experimental stenotic model [30] and circumstantial observations in stenotic carotid arteries [44] may be more relevant to our present work than the studies of cerebrovascular or coronary flow reserve. Interestingly, already 3 decades ago Alexandrov et al. [44] suggested that the diameter of carotid stenosis (angiographic NASCET) may consistently underestimate the “true” hemodynamic stenosis severity. Rather, the highest PSV in the stenotic segment would provide a more accurate measurement of the carotid stenosis hemodynamic significance [44]. This idea is in perfect keeping with our present findings as the flow velocities incorporate the LL effect (Figure 4). Overall, results from the present study are consistent with a number of earlier circumstantial observations that, surprisingly, did not lead in the past to investigating the hemodynamic effect exerted by the length of carotid stenotic segment. Lack of such investigations is striking, particularly as the role of carotid LL has not been ignored in several other clinically relevant aspects [92,93,94].

### 4.10. Classic DUS Interpretation Can Mislead Clinical Decisions: Evidence from Angiography

Although catheter angiography has been considered the gold standard for evaluating the severity of carotid artery stenosis, due to (small but non-negligible) risks related to the procedure, in current clinical practice the invasive angiogram—unless part of the therapeutic procedure [1,4,95,96,97,98,99,100]—is usually reserved for select diagnostic situations [101]. The downside is inappropriate clinical decisions as non-invasive techniques are documented to have significant problems with determining stenosis characteristics to correctly guide stenosis severity-based treatment choices [28,95,96,102,103]. Specifically, DUS may be reasonably accurate at discriminating between the presence or absence of a “significant” cross-sectional carotid artery stenosis (i.e., <50% or 50% to 99%), supporting the use of DUS as the first-choice modality for the detection of carotid stenosis [101]. However, DUS fails in stenosis category subclassification [18,25,27,28,29,101]. The latter, given the continued guideline focus on cross-sectional lesion severity thresholds for intervention [13,14,15,16], is particularly relevant in clinically asymptomatic patients—a cohort including those who (if left untreated) may suffer from a future carotid-related stroke [2,7,104]. The Buffalo Group found a relatively high (80%) positive predictive value of carotid DUS for identifying appropriate symptomatic (angiographic stenosis ≥50%) patients for carotid intervention, with false-positive proportion of 20% [102]. However, DUS significantly failed in identifying appropriate asymptomatic candidates for carotid intervention (positive predictive value for angiographic stenosis ≥ 60% of only 59%, with a false-positive value of 41%) [102]. They concluded that with the DUS demonstration of stenosis severity, an inappropriate surgical intervention (carotid endarterectomy, CEA) would occur (in the absence of catheter angiography verification) in one of every five patients with DUS studies performed exclusively in the best ultrasound laboratories [102]. Recent evidence demonstrates that computed tomography angiography (CTA)—even with the use of NASCET (i.e., correct [16,65]) measurement technique rather than the more routine calculation of area stenosis [96,103])—may fail to resolve this critical clinical issue [28,95]. Data by Hejnenbrok-Kal and colleagues [105] demonstrate that the harm of performing angiographic verification in false-positive DUS cases may be much smaller than the harm of performing unnecessary endarterectomy. Catheter angiographic verification demonstrates a poor diagnostic accuracy of contemporary carotid CTA in a recent series of cases reporting 50–70% NASCET stenosis (positive predictive value of only 0.29, *p* < 0.001), indicating that “*a significant number of patients referred to CEA may undergo unnecessary surgery and may become exposed to associated potential complications*” [95]. Importantly, the room for fundamental errors in relation to stenosis severity below the procedural indication cutoff is minimized with revascularization techniques that involve, as a routine part of the procedure, angiographic verification of stenosis severity [1,4,95,96,97,98,99,100]. Contemporary evidence shows that angiographic verification of stenosis severity as the first step of the revascularization procedure results in aborting the intervention (due to the absence of stenosis severity indication) in ≈20% patients scheduled for revascularization on the basis of non-invasive imaging [97,102]. For these reasons, some recent suggestions to bring the old, failed concept of performing carotid surgery on the basis of DUS imaging alone (i.e., in the absence of any verification with non-invasive or catheter angiography) [106,107,108,109] back to contemporary clinical practice [110] need to be considered surprising. The apparent “safety” of the procedure cannot be accepted as replacement for a (missing, in a proportion of patients) indication. According to recent analysis of 171,816 CEAs, one in every five patients may be undergoing CEA based on DUS alone [110]. The finding that “*performing CEA for asymptomatic bifurcation stenosis based on DUS alone is a safe practice which achieves clinically equivalent perioperative and long-term freedom from cerebral ischemia and mortality relative to CEA based on advanced imaging*” may be undisputable, but it is surprising to see this used—in this scenario—as an argument to recommend CEA on the basis of DUS alone as CEA, in a significant proportion of patients, may be an unnecessary procedure [17,24,26,27,102]. A review of current practice shows the use of 13 distinct (PSV, EDV and ratios) to grade >80% stenosis [111], consistent with suboptimal accuracy of classic DUS interpretation in grading carotid stenosis severity. Indeed, vast discrepancies between contemporary CTA and DUS were found in a recent series of consecutive patients, so that 45.5% of those with carotid artery stenosis of 50–69% DUS had a significantly different stenosis severity by NASCET-CTA [27]. This is consistent with our previous work showing significant imaging modality-dependent variations in carotid cross-sectional stenosis severity against IVUS [45] used as the gold standard in vascular imaging [46,47,48]. Overall, it is clear today that particularly the patients with carotid artery stenosis severity of “50% to 69%” by DUS require proper attention to minimize the potential diagnostic error of DUS [101]. Our present work indicates that at least part of the DUS error in determining cross-sectional severity may arise from the effect of lesion length on PSV and EDV. Carotid LL should be taken into consideration when developing algorithms that enhance carotid ultrasound image interpretation quality and machine learning models to classify stenosis severity for automated generation of ultrasound report [112,113]. While the present study provides corrective formulas that include the LL effect on Doppler flow velocities, resulting in an increased precision of cross-sectional stenosis severity determination (Graphical Abstract), DUS operators should continue to be sensitive to other confounders, such as contralateral stenosis or occlusion, cardiac output, blood volume and pressure, and the effect of vessel compliance/patient age [30,34,35].

### 4.11. Strengths and Limitations

In a large sample of quality data [114] in consecutive patients presenting with a “significant” carotid stenosis, we have performed a systematic evaluation of the thus-far neglected effect of the stenotic segment length on lesional flow velocities. We found that the LL effect is significant statistically and relevant clinically; a phenomenon that is grounded in theoretical fluid dynamics and in prior experimental work in a stenotic flow model [30]. One limitation of the present study is that we have not investigated the role played by the stenosis entrance angle; a parameter recently suggested to modulate the LL effect in its relation to abnormal trans-lesional functional flow reserve in the coronary tree [84]. Similarly, we have not detaily evaluated the potential impact of distal lumen expansion and the pressure drop due to post-stenotic turbulence marked by bruit or murmur [30]. Any precise determination of the carotid stenosis entrance angle and the course of the distal lumen expansion may be unfeasible when using planar angiographic images, as this angle may not only vary in the different planes but also it may be further affected by the angle of the angiographic imaging plane. Our use of the length of segment with ≥50% diameter stenosis showed a qualitative effect of LL on PSV/EDV similar to that of total LL (Figure 3), indicating that the lesion entrance/exit phenomena may play a rather limited role in the clinically relevant LL contribution to PSV/EDV.

Our present use of angiography for measurement of lesion length but use of IVUS for MLA determination might be considered, by some, a limitation. Nevertheless, it needs to be pointed out that while IVUS fails in any precise measurement of the carotid stenotic segment length (because of the IVUS transducer “jumping” artifact with systole/diastole [50]), angiography is generally less accurate than IVUS in determination of cross-sectional stenosis severity (Figure 5). For these reasons our combining of an accurate technique for cross-sectional stenosis severity determination with an adequate technique for determination of carotid lesion length [44,45] may, in fact, represent an important strength of the present study.

The flow through the curved carotid arteries is laminar but helical [115]. In the presence of helical flow, the validity of the Doppler angle correction may be questionable, although universally used. Rather than testing the consistency of velocity measurements taken from a selected location at a variety of acceptable angles to determine whether the resulting velocity value is the same if the flow is observed from different angles, consistent with routine clinical practice we accepted Doppler angles between 45° and 60°. In carotid stenosis, residual lumen diameter may be estimated from bruit frequency [116]. Thus, another potential limitation of our work is lack of analysis of proportion of patients presenting with carotid bruit. Although carotid bruit may not be a reliable sign of significant extracranial carotid artery disease (positive predictive value of 37%, false negative rate of 43%, normal carotid arteries in 32% of patients with a bruit [117,118]), a large-scale meta-analysis suggested that auscultation for carotid bruits in patients at risk for atheroscleritic heart disease could help select those who might benefit from an aggressive modification of overall atherosclerotic cardiovascular risk [119].

It needs to be noted that our IVUS measurements were performed in diastole. Work by the Ramnarine group showed a mean absolute diameter change of the stenotic carotid artery from diastole to systole of 0.45 mm (absolute change by 6.9%) [120]. As a result of the Venturi effect, there is significant diametric reduction during systole due to the pressure drop within the stenotic segment lumen [121], affecting local hemodynamics and thus potentially the PSV/EDV measurement.

While the present analysis excluded patients with contralateral carotid stenosis ≥50% or occlusion that may be associated with increased flow velocities in the index artery [40,41], we have been unable to correct for potential confounders of differences in cardiac output [34] and vessel compliance [35]. Although such potential corrections could lead to a “cleaner” data set, we wanted to root our analysis in clinical relevance that does not routinely correct flow-velocity-based estimation of cross-sectional stenosis severity for cardiac output and vessel compliance. Still, with our clinical practice—focused approach, we did find a clear and statistically significant effect of LL on PSV and EDV (Figure 3 and Figure 4).

Last but not least, it needs to be noted that as the LL and MLA information is missing on routine first-line Doppler examination, our present analysis is “backward” (i.e., the LL and MLA data were obtained subsequently to Doppler examination). This, however, cannot be considered a limitation in raising awareness, amongst the physicians and cardiovascular technologists performing DUS examinations, of the LL contribution to PSV and EDV. A further large-scale study is needed to evaluate, apart from the catheter angiographic and IVUS measurements (as used in the present study), the value of taking LL from DUS in order to correct the PSV and EDV estimation of cross-sectional severity for LL using presently developed mathematic formulas. Ideally, the magnitude of LL effect in specific lesion length intervals ≥7 mm needs to be further elucidated. Finally, because of the IVUS limitation in determining lesion length at carotid bifurcation resulting from a marked systolic-diastolic “jumping” of the probe, we measured angiographic LL in the projection best presenting stenotic segment length with minimal foreshortening. Possibly, a more exact LL measurement would be taken from CTA. Nevertheless, CTA acquisition is no longer part of routine imaging in our all our patients while we aimed for the study data to be applicable to routine clinical practice. A further, yet larger-scale multicentric study will need to evaluate the exact proportion of clinically symptomatic and clinically asymptomatic all-comer patients in whom determination of the LL effect (on PSV/EDV) changes their further diagnostic and management pathway [96]. A larger-scale data set is needed to precisely evaluate the diagnostic accuracy of LL-corrected PSV and EDV values for the specific clinically–relevant diameter stenosis severity thresholds/intervals (such as ≥50%, ≥70%, or ≥80%).

## 5. Conclusions

Despite a growing understanding of the role played by lesion morphology, according to current guidelines the cross-sectional stenosis severity determines—along with the presence/absence of clinical symptoms of cerebral ischemia—the treatment decisions in patients with symptomatic and asymptomatic carotid stenosis.

This study provides, for the first time, systematic evidence that carotid longitudinal lesion length affects PSV and EDV in addition to the well-known effect exerted by cross-sectional stenosis severity. For carotid lesions causing ≈50–90% cross-sectional stenosis, there is an increase in PSV and EDV with an increase in lesion length. The lesion-length contribution to PSV and EDV may in part explain the observed “inaccuracy” of Doppler velocities in determining cross-sectional stenosis severity; an effect that becomes significant with lesion lengths ≥7 mm. There is a systematic lesion length–dependent overestimation of cross-sectional severity, and the magnitude of (correctible) error increases with increasing stenotic segment lengths (Graphical Abstract).

We determined equations that incorporate both lesion length and cross-sectional stenosis severity in Doppler velocities and simple mathematic formulas that correct the velocity-dependent estimation of cross-sectional stenosis severity for the effect of stenotic segment length (Graphical Abstract). Taking into account the contribution of lesion length to the values of lesional PSV and EDV may improve the precision of cross-sectional stenosis severity assessment using Doppler velocities.

Physicians and cardiovascular technologists performing transcutaneous Doppler ultrasound evaluation of carotid stenoses—as well as those who use the PSV/EDV data as a basis for decisions regarding their patients’ further diagnostic and management pathway—should be aware that the PSV and EDV “numeric value [in m/s or cm/s]” includes, beyond the component of cross-sectional stenosis severity, the component of lesion length, so that (1) the actual cross-sectional stenosis severity may be lesser that that conventionally inferred from PSV (EDV), and (2) the greater the lesion length, the greater its contribution to PSV (EDV), starting with stenotic segment lengths ≥7 mm. As lesion length can be usually estimated already at the stage of DUS examination, we advocate including stenosis length measurement in DUS reports.

## Figures and Tables

**Table 1:** Clinical characteristics of the study patients (n = 293).

Age, years	66 (60–72)
Gender = men, n (%)	188 (64.2)
Symptomatic, n (%)	187 (63.8)
Diabetes, n (%)	94 (32.1)
Insulin, n (%)	31 (10.6)
Smoking (current or past), n (%)	156 (53.2)
Arterial hypertension, n (%)	260 (88.7)
on hypotensive pharmacotherapy	260 (88.7)
SBP, mmHg	132 (125–137)
DBP, mmHg	78 (70–84)
h/o myocardial infarction, n (%)	74 (25.2)
CAD, n (%)	196 (66.9)
PAD, n (%)	44 (15.0)
BMI, kg/m^2^	27.7 (25.7–30.1)
Creatinine, μmol/L	85 (74–101)
eGFR < 60, mL/min, n (%)	65 (22.2)

Data are given as median (Q1–Q3) or number (n, %). CAD—coronary artery disease, PAD—peripheral artery disease, BMI—body mass index, h/o—history of, SBP—systolic blood pressure, DBP—diastolic blood pressure, eGFR—estimated glomerular filtration rate.

**Table 2:** Characteristics of the index carotid lesions (n = 293).

PSV (m/s)	2.5 (1.9–3.3)
EDV (m/s)	0.9 (0.6–1.2)
(total) LL (mm)	10.5 (7.2–14.4)
LL ≥ 50% DS (mm) ^#^	2.4 (1.5–3.7)
QA-DS (NASCET, %)	59.0 (54.0–67.0)
QA (circumferential) MLA (mm^2^)	3.3 (2.3–4.4)
QA_DENSITOM_-MLA (mm^2^)	5.9 (4.2–7.7)
IVUS-MLA (mm^2^)	6.1 (4.6–8.4)

Data are provided as median (Q1–Q3) ^#^ The length of the segment with lesion exceeding 50% diameter stenosis by quantitative angiography, DS—diameter stenosis, EDV—end-diastolic velocity, IVUS—intravascular ultrasound, LL—(total) lesion length, MLA—minimal lumen area, NASCET—North American Symptomatic Carotid Endarterectomy Trial method [43], PSV—peak systolic velocity, QA—conventional (planar) quantitative angiography, QA_DENSITOM_—contrast column density-based quantitative angiographic measurements.

## Data Availability

Part of the data were communicated to the Transcatheter Cardiovascular Therapeutics (TCT, W. Dabrowski, Oral Presentation). Data on file will be made available from the corresponding authors on reasonable request.

## Supplementary Materials

The following supporting information can be downloaded at: https://www.mdpi.com/article/10.3390/diagnostics15101259/s1, Figure S1: Angiographic verification of carotid stenosis severity in the Graphical Abstract Patient 1 (A) and Patient 2 (B). The content of the supplementary material is the detailed description of the Graphical Abstract and Supplementary Figure S1.

## Abbreviations

The following abbreviations are used in this manuscript:

CAD	Coronary Artery Disease
CEA	Carotid Endarterectomy
CT	Computed Tomography
CTA	Computed Tomography Angiography
DENSITOM	Densitometric
D_min_	Minimal Lumen Diameter
DS	Diameter Stenosis
DUS	Duplex Ultrasound
eGFR	Estimated Glomerular Filtration Rate
EDV	End-Diastolic Velocity
IVUS	Intravascular Ultrasound
LL	Lesion Length (unless specified otherwise: total lesion length)
MLA	Minimal Lumen Area
NASCET	North American Symptomatic Carotid Endarterectomy Trial (method)
PAD	Peripheral Arterial Disease
PSV	Peak-Systolic Velocity
QA	Quantitative Angiography
RD	Reference Diameter
V	(flow) velocity
