# Supplementary material for: The Effect of Lesion Length on Doppler Velocities Used Routinely to Determine Carotid Stenosis Cross-Sectional Severity
Source: Diagnostics (Basel). 2025 May 15;15(10):1259. doi: 10.3390/diagnostics15101259 (PMC12110091; doi:10.3390/diagnostics15101259)
Supplement: Supplementary file 1 [file diagnostics-15-01259-s001.zip › diagnostics-3460242-supplementary.pdf]

Detailed description of the Graphical Abstract

Transcutaneous Doppler ultrasound is a fundamental technique in evaluating carotid stenosis cross-sectional severity in clinical practice because the stenotic peak-systolic and end-diastolic velocities (PSV and EDV) relate to angiographic diameter stenosis. In a cohort of nearly 300 consecutive patients with carotid stenosis presenting for potential revascularization, this study established a significant contribution of the lesion length (LL) component to PSV and EDV. We found that the longer the lesion the greater the flow velocity; an effect consistent with fluid dynamics and statistically significant for carotid lesions ⩾7 mm in length. This fundamental finding may explain, at least in part, the observed 'inaccuracy' of flow velocities in determining the cross-sectional stenosis severity of carotid stenosis.

The Graphical Abstract presents an example of two clinically asymptomatic patients who exhibited similar Doppler spectra in index internal carotid arteries (PSV 3.92 m/s and EDV 1.27 m/s; reference diameter, RD, 5 mm in both). According to Grant criteria (Society of Radiologists in Ultrasound Consensus, 2003 [23]), the PSV value of 3.92 m/s is indicates >70% cross-sectional diameter stenosis (DS; >80% DS according to Bluth criteria [21]). As per carotid stenosis management guidelines [13-16], the finding of stenosis severity >70-80% determines further diagnostic and therapeutic steps.

We established that the relationship between flow velocities, LL and minimal lumen area (MLA) is defined by the following formulas: PSV = 0.31 × LL/MLA + 2.02 [m/s] and EDV = 0.12 × LL/MLA + 0.63 [m/s]. Thus incorporating the LL effect on PSV, the DS estimation is 75.4% in Patient 1 but 'only' 54.2% in Patient 2 ($MLA = \pi (D_{min})/2)^2$; $DS = [(RD-D_{min})/RD] \times 100\%$).

EDV–based evaluation of DS taking into account the stenotic segment length indicated, similarly, 73.9% (Patient 1) and 51.0% (Patient 2).

The Doppler velocity–based estimation of DS incorporating the LL effect was consistent with quantitative angiographic measurements that showed 73.6% DS in Patient 1 and 53.1% DS in Patient 2 (for full-size angiograms see Suppl. Fig 1 below).

When planning management of patients with carotid stenosis, the lesion length contribution to observed flow velocities needs to be taken into consideration. Note that Patient 2, despite the PSV/EDV values suggesting a tight cross-sectional stenosis does not meet the current "% diameter stenosis" – based guideline criteria for potential interventional management. This is relevant as some would continue to recommend, today, carotid endarterectomy in absence of lesion severity verification [109,110]. The problem may not be resolved by performing further non-invasive angiographic evaluation as this may fail to rectify the diagnostic information [97-99]. Importantly, the endovascular route of revascularization encompasses – as the initial procedural step – angiographic verification of the stenosis degree, so that the procedure is abandoned in absence of the guideline-indicated cross-sectional stenosis severity as a fundamental requirement.

Abbreviations: DS – diameter stenosis, EDV – end-diastolic velocity, LL – lesion length, MLA – minimal lumen area, PSV – peak-systolic velocity, D_{min} – minimal lumen diameter, RD – reference diameter

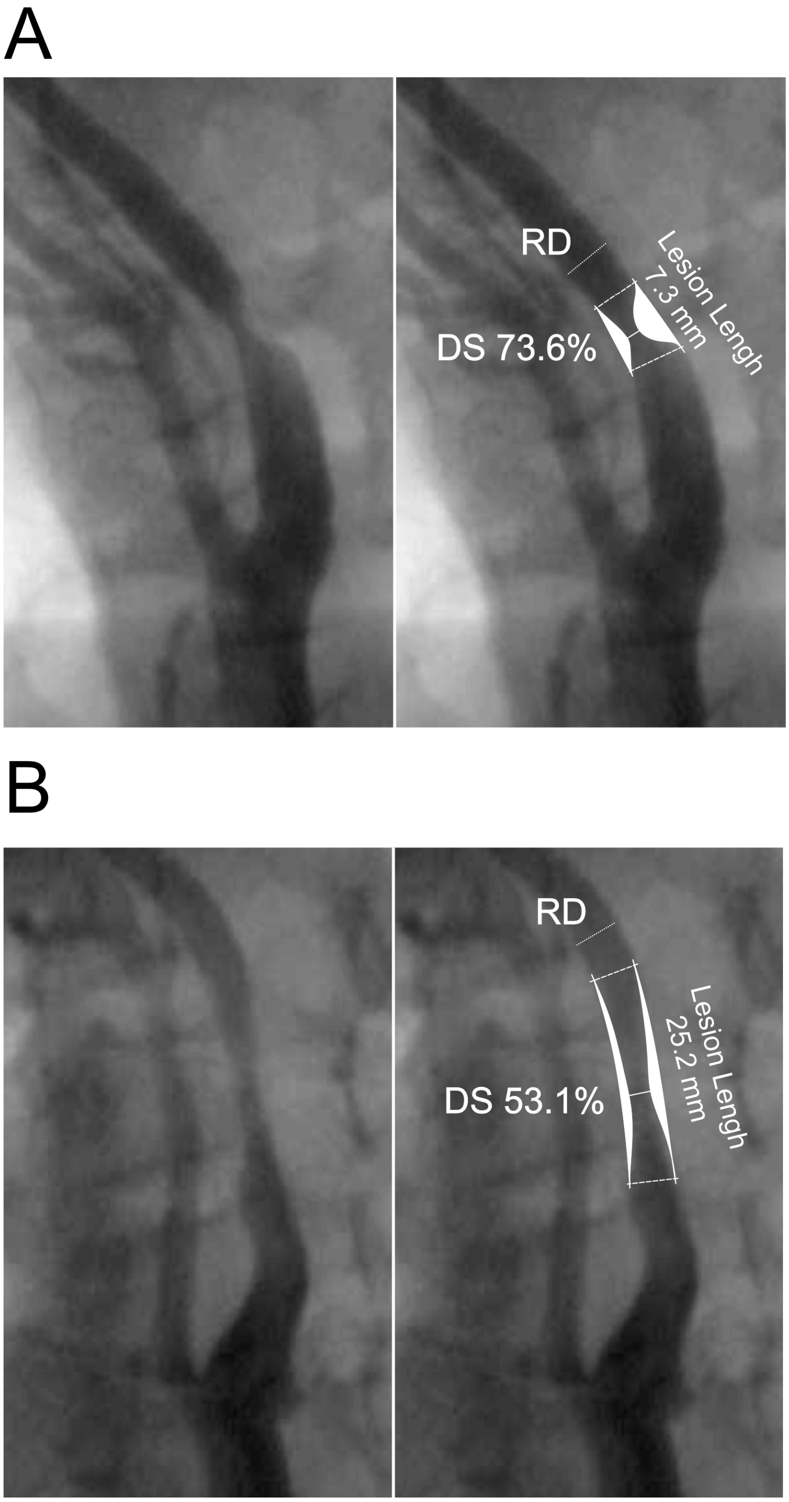

Suppl. Figure S1. Angiographic verification of carotid stenosis severity in the Graphical Abstract Patient 1 (A) and Patient 2 (B).

Full-size raw angiograms are presented on the left; on the right are quantitative angiography measurements of the cross-sectional % diameter stenosis (DS) and lesion length. Note that because of the lesion length effect on PSV and EDV as identified and documented in the present study, the "same" values of PSV and EDV can be associated with different degrees of the stenosis cross-sectional severity ("% DS") that determine further, guideline-indicated, patient management pathway. See text for formulas correcting the PSV (EDV)-estimation of cross-sectional stenosis severity for the effect of lesion length. RD – Reference Diameter.
